# Methicillin-Resistant Staphylococcus Aureus: A Very Rare Cause of Meningitis

**DOI:** 10.7759/cureus.10370

**Published:** 2020-09-10

**Authors:** Maham A Mehmood, Madanmohan Patel, Harshavardhan Sanekommu

**Affiliations:** 1 Internal Medicine, BronxCare Hospital, New York, USA; 2 Internal Medicine, American University of the Caribbean School of Medicine, Bronx, USA

**Keywords:** methicillin resistant staphylococcus aureus (mrsa), lumber puncture, meningitis, vancomycin, bacteremia

## Abstract

Community-acquired methicillin-resistant Staphylococcus aureus (CA-MRSA) is mostly implicated in soft tissue and skin infections. Cases with meningitis caused by CA-MRSA are rare. High index of suspicion should be kept for physicians as bacterial meningitis is a medical emergency and if untreated, has a high mortality rate. Urgent steps need to be taken to determine the cause and implement therapy. Here, we reported a case of a 58-year-old female with MRSA bacteremia and meningitis as confirmed by positive blood cultures and cerebrospinal fluid analysis; successfully managed with vancomycin and rifampin.

## Introduction

Staphylococcus aureus is rampant and a widely distributed microorganism. It is one of the most important pathogens of nosocomial and community-acquired infections. Staph aureus causes a wide variety of diseases. The first methicillin-resistant staph aureus (MRSA) was discovered by Jevons in the United Kingdom and it is now seen worldwide. The clinical and bacteriological properties of community-acquired methicillin-resistant Staphylococcus aureus (CA-MRSA) are different from those of hospital-acquired MRSA [1]. Despite its increasing distribution, meningitis continues to be associated with serious and occasionally fatal outcomes. Treatment is difficult because of the critical location of these infections and the blood-brain barrier, which limits the penetration of systemically administered antibiotics to the site of infection [2]. This paper emphasizes the need for early recognition and interventions as soon as meningitis is suspected, while identifying a case where synergistic effects of antibiotics are utilized to improve the penetration of antibiotic therapy for adequate treatment.

## Case presentation

A 58-year-old female with a medical history that includes hypertension and asthma presented to the ED with gradual onset altered mental sensorium of two days duration. She further endorsed active intravenous heroin usage and the last dose was on the day of arrival to the hospital. The patient partially responded to narcan en route to the hospital. On presentation, the patient was noted to be alert and oriented to self only, she was afebrile with temperature of 99.2F, pulse 106/min respiratory rate 16 /min, blood pressure 122/70 mm HG and oxygen saturation of 96% on room air. Cardiovascular respiratory and gastrointestinal systems were reported normal, no focality and signs of meningism was noticed on neurological examination.

Pertinent laboratory findings included WBC 19.9 k/ul, hemoglobin 10.9 g/d, platelets 375k/ul erythrocyte sedimentation rate >120mm/hr, C-reactive protein 212mg/L, HIV-negative, drug screen was positive for cannabis and opiates and chest x-ray revealed diffuse reticulonodular interstitial thickening, compatible with endobronchial infection versus atypical infectious process. All other labs including renal profile, liver function test and computed tomography (CT) of head were entirely normal. The patient was admitted to the floor for acute encephalopathy secondary to opiate overdose versus septic encephalopathy due to possible pneumonia (COVID vs community-acquired). She was started on broad-spectrum antibiotics (vancomycin 1.25 g/twice a day, cefepime 1 g/thrice a day, doxycycline 100 mg/twice a day) and intravenous fluids. The clinical course of the patient was complicated with the development of high-grade fever, hypoxia and worsening of mentation and she was transferred to the critical care unit. Acyclovir and ampicillin were added to the current regimen with a suspicion of meningoencephalitis. The blood culture grew gram-positive cocci which later turned out to be MRSA and pneumonia workup (SARS-COV, mycoplasma, legionella, and streptococcus) was negative. Seven days into her illness, the patient became completely oriented and mental status has improved, but remained persistently bacteremic and febrile despite being on vancomycin therapy. The patient and the family continued to refuse lumbar puncture. The transesophageal echocardiography did not reveal any vegetations and the heart function was normal otherwise. After rigorous counselling, patient agreed for lumbar puncture (10 days into her illness) and the results were confirmatory for bacterial meningitis with gram stain positive for gram-positive cocci and bacterial antigen for Staphylococcus aureus (as seen in Table 1).

**Table 1:** CSF results after 10 days of broad-spectrum antibiotics CSF, cerebrospinal fluid; RBC, red blood cell; WBC, white blood cell; PCR, polymerase chain reaction

Spinal Fluid	Value	Reference Range
Appearance	Clear and colorless	
Glucose	22 mg/dL	40-70 mg/dL
Protein	835 mg/dL	15-45 mg/dL
Lactic Acid	5.0 mmoles/L	(0.6-2.2 mmoles/L)
WBC count	65	
RBC Count	13	
Lymphocytes	12%	
Neutrophils	84%	
Monocytes	4%	
Serology/Microbiology/Virology		Results
Gram stain		Gram-Positive Cocci
Bacterial Antigen		Positive for S. aureus
Herpes Simplex Virus 1 and 2 PCR		Negative
Cytomegalovirus PCR		Negative
VRDL		Nonreactive
Cryptococcal Antigen		Negative
Aerobic Culture		No growth

**Figure 1:**
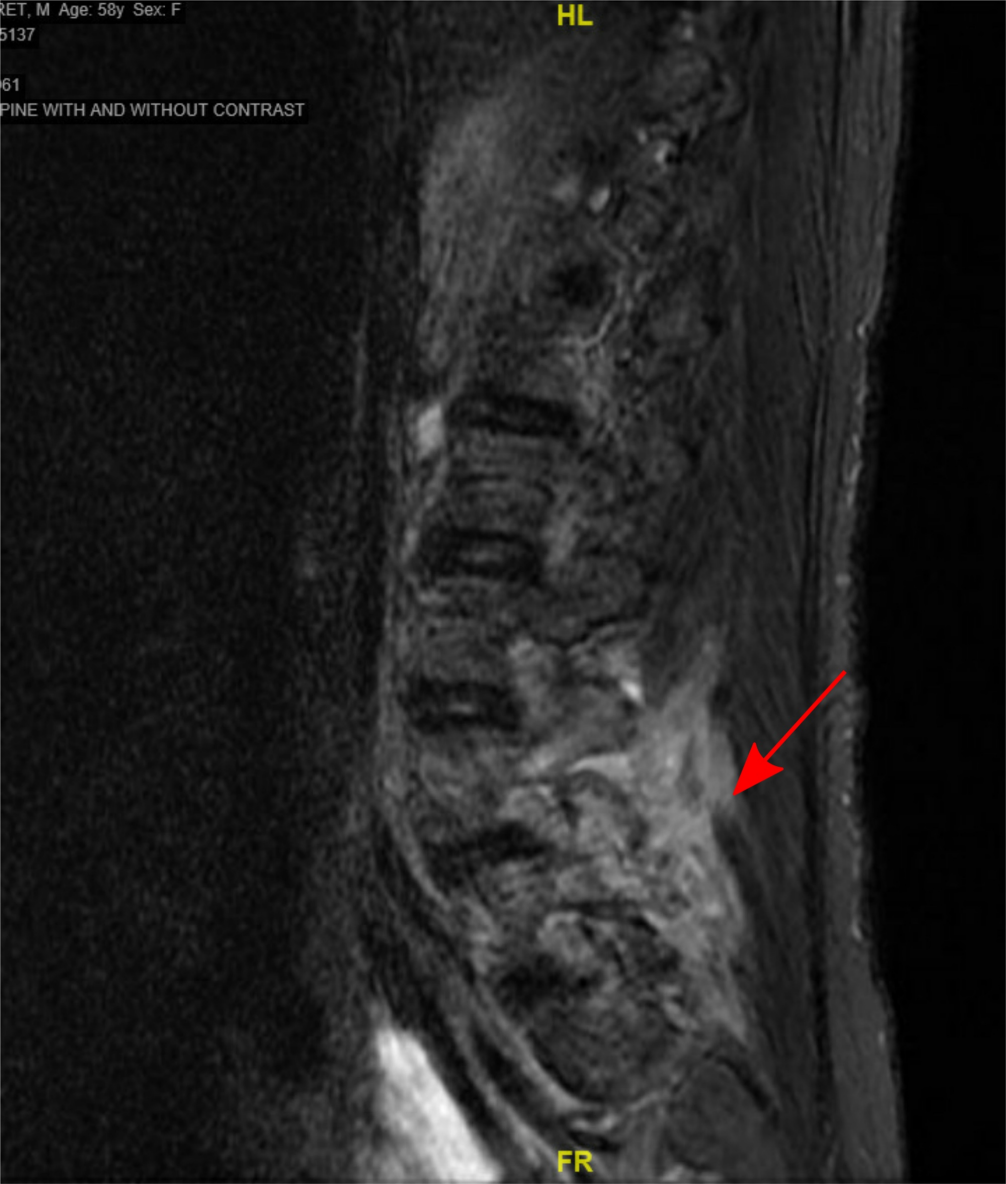
MRI of the lumbar spine showing epidural abscess (red arrow)

Infectious disease specialty was called on board and treatment failure was considered by cause of subtherapeutic levels of vancomycin, hence the patient was loaded with vancomycin 2 g and then kept on 1.5 g/twice a day and rifampin 900 mg/twice a day was added for a synergistic effect. All other antibiotics were discontinued. At this point, the patient developed lower back pain, flank pain and dysuria. CT abdomen and pelvis and urine analysis were consistent with acute pyelonephritis and the patient was started on ceftriaxone. The symptoms improved, but the back pain remained. Magnetic resonance imaging of the lumbar spine (MRI LS) revealed posterior epidural phlegmon at L4-5, causing severe spinal stenosis. L4 and L5 spinous process edema also noted with T1 signal loss with reference to osteomyelitis (as seen in Figure 1). Neurosurgery was taken on board and the patient underwent bilateral L4-L5 lumbar laminectomy, removal of epidural abscess and granulation tissue with decompression of the nerve root. Aerobic cultures were also sent which were negative, most likely due to the patient being on antibiotics for a prolonged duration.

Fifteen days into her illness, the patient had the first negative blood culture, remained afebrile and improved clinically. The patient was discharged to short-term rehabilitation to complete a total of six weeks of vancomycin and two weeks of rifampin. The patient was followed up at a six-week interval, she remained afebrile with persistently negative blood cultures, and she had regained her physical strength as well and was successfully discharged home.

## Discussion

Community-acquired bacterial meningitis in adults in developed countries is largely attributed to: Streptococcus pneumoniae, Neisseria meningitidis, and Listeria monocytogenes (primarily in patients over 50 years of age, or those who have deficiencies in cell-mediated immunity) [3]. Meningitis caused by Staph Aureus is extremely rare, with an annual incidence of 1%-3% [4]. Of these cases, more than 50% are associated with a neurosurgical intervention where the bacteria are introduced during procedures. The rest are due to spontaneous or community-acquired meningitis, most likely due to infection outside of the central nervous system (CNS). To our knowledge, epidemiological data for MRSA meningitis has not been widely reported because of its rarity. 

The classic triad of acute bacterial meningitis consists of fever, nuchal rigidity, and a change in mental status, which occurs in 41% of patients. The most common clinical features include a severe headache (84%), fever greater than 38°C (74%), stiff neck (74%), a Glasgow Coma scale <14 (71%), and nausea (62%) [5]. The clinical and laboratory findings of bacterial meningitis overlap with those of meningitis caused by viruses, mycobacteria, fungi, or protozoa [6]. Differentiation of these pathogens from bacterial meningitis requires careful examination of cerebrospinal fluid (CSF) parameters, neuroimaging, when indicated, as well as consideration of any epidemiologic factors that would raise the possibility of specific bacterial or non-bacterial CNS infections.

Initial investigations of suspected meningitis should include blood tests including a complete blood count with differential and platelet count and two aerobic blood cultures of appropriate volume (ideally, prior to the initiation of antimicrobial therapy). Empiric therapy of broad-spectrum antibiotics should be started immediately, without waiting for the lab results. Serum electrolytes and glucose, blood urea nitrogen, and creatinine concentrations are helpful in determining the CSF-to-blood glucose ratio. Coagulation studies are indicated, especially if petechial or purpuric lesions are noted. Blood cultures are often positive and can be useful in the event that CSF cannot be obtained before the administration of antimicrobials. Approximately 50% to 90% of patients with bacterial meningitis have positive blood cultures [5].

Every patient with suspected meningitis should have CSF obtained unless a lumbar puncture (LP) is contraindicated [7]. A computed tomographic (CT) of head scan is sometimes performed before LP to exclude a mass lesion or increased intracranial pressure, which rarely leads to cerebral herniation during subsequent CSF removal [7]. However, a screening CT scan is not necessary in the majority of patients and should not delay the initiation of antibiotics. The antibiotics should be preferably started after the blood cultures are drawn.

The exact management for MRSA meningitis infection is still unknown and there are no current established guidelines for treatment. Given significant rates of MRSA, vancomycin (15 to 20 mg/kg IV every 8 to 12 hours depending on) should be used as initial therapy when S. aureus is suspected or proven. The exact duration of therapy is based on clinical response and is mostly administered for 10 to 14 days [8,9]. The Infectious Diseases Society of America has recommended two weeks of vancomycin therapy for MRSA meningitis. The major limitation of vancomycin therapy is its poor penetration into CSF estimated penetration of 1% and 5% with uninflamed and inflamed meninges, respectively [10]. In our literature research, we found out that rifampin may be beneficial as it achieves bactericidal concentrations in the CSF, regardless of meningeal inflammation, and can be used as a form of treatment. The recommended dose was 600 mg orally or IV once daily or 300 to 450 mg twice daily [11,12].

We implemented our research findings on our patient with positive outcomes and can safely recommend that rifampin can be added to vancomycin for a combined synergistic effect. In our research, we ascertained, that based upon some case reports and case series of patients with MRSA meningitis, alternatives to vancomycin include linezolid (600 mg IV twice daily) [12], TMP-SMX (5 mg/kg of the trimethoprim component IV every 8 to 12 hours) [9] and daptomycin (6 to 10 mg/kg IV once daily) usually combined with rifampin [13]. Further studies are needed to establish the benefit of these agents for the treatment of meningitis.

Patients with persistent bacteremia should be reevaluated for adequate therapy and other sources of infection, as seen in this patient. After repeated positive blood cultures, we evaluated the therapy and made changes accordingly. Also, we followed up with the physical exam findings to determine a source of infection. This investigation led us to conduct an MRI of the spine which revealed an epidural abscess. Suspected seeding of bacteria from abscess leads to bacteremia and meningitis. We addressed the abscess as a possible source of infection through surgical intervention and the antibiotics.

## Conclusions

Our case emphasizes the importance of keeping a high index of suspicion for MRSA meningitis and disseminated infection in IV drug users. MRSA meningitis is very rare and can be easily missed. We must emphasize the importance of thorough physical examination and clinical evaluation, particularly in patients with persistent bacteremia to identify the source of bacteremia. Bacteria can seed any organ, and the consequences can be lethal and devastating. Good clinical acumen, timely diagnosis, initiation of effective therapy and interventions can be lifesaving.
